# Oligomerisation of *C. elegans* Olfactory Receptors, ODR-10 and STR-112, in Yeast

**DOI:** 10.1371/journal.pone.0108680

**Published:** 2014-09-25

**Authors:** Muhammad Tehseen, Chunyan Liao, Helen Dacres, Mira Dumancic, Stephen Trowell, Alisha Anderson

**Affiliations:** 1 CSIRO Food Futures National Research Flagship & CSIRO Ecosystem Sciences, Australia, Canberra, ACT, Australia; 2 Center for Desert Agriculture, Division of Biological and Environmental Sciences and Engineering, King Abdullah University of Science and Technology, Thuwal, Kingdom of Saudi Arabia; Monell Chemical Senses Center, United States of America

## Abstract

It is widely accepted that vertebrate G-Protein Coupled Receptors (GPCRs) associate with each other as homo- or hetero-dimers or higher-order oligomers. The *C. elegans* genome encodes hundreds of olfactory GPCRs, which may be expressed in fewer than a dozen chemosensory neurons, suggesting an opportunity for oligomerisation. Here we show, using three independent lines of evidence: co-immunoprecipitation, bioluminescence resonance energy transfer and a yeast two-hybrid assay that nematode olfactory receptors (ORs) oligomerise when heterologously expressed in yeast. Specifically, the nematode receptor ODR-10 is able to homo-oligomerise and can also form heteromers with the related nematode receptor STR-112. ODR-10 also oligomerised with the rat I7 OR but did not oligomerise with the human somatostatin receptor 5, a neuropeptide receptor. In this study, the question of functional relevance was not addressed and remains to be investigated.

## Introduction

G-protein coupled receptors (GPCRs) are the largest and most diverse superfamily of proteins and are present in every eukaryotic cell [1]. They are involved in the senses of vision, smell, taste, pain, cell recognition and communication processes. GPCRs are characterised structurally by an amino-terminal extracellular domain, a carboxyl-terminal intracellular domain and seven hydrophobic transmembrane domains. They are activated by a wide variety of ligands, including peptide and non-peptide neurotransmitters, hormones, growth factors and odorant molecules and are encoded by the largest gene family in most animal genomes. For example, 1% of the total genes in *Drosophila*, ∼4% of all genes in the human genome and >5% of all genes in *Caenorhabditis elegans* encode GPCRs [2].

GPCRs were once thought to act as monomers but in the last decade evidence has emerged that the primary signalling unit consists of homo- or heterodimers [3]–[6]. GPCR oligomerisation is also important for receptor maturation, trafficking, agonist specificity and signalling [7], [8]. The potential diversity of GPCR heterodimers can increase the repertoire of GPCR recognition and signalling via allosteric mechanisms [9], [10]. For example, surface expression of α2C- and α1D-adrenergic receptors is greatly enhanced by co-expression of β2-adrenergic receptors [11]–[13], while the GABA_B_ receptor subunits R1 and R2 associate, through their cytoplasmic tails, in the endoplasmic reticulum and are targeted to the plasma membrane as a preformed dimer, independent of agonist regulation [14]. It has been shown that hetero-oligomerisation can either inhibit or facilitate endocytosis and affect receptor signalling [15], [16]. For example, hetero-oligomerisation between the beta-1 adrenergic receptor (β1-AR), which is poorly internalised, and the β2-AR, which is strongly internalised, results in inhibition of agonist-promoted internalisation of the latter.

Although evidence for GPCR hetero-oligomerisation is mostly drawn from studies on a few, well-characterised vertebrate receptors for hormones and neurotransmitters, the largest families of GPCRs are involved in chemoreception. Vertebrate olfaction and taste perception (sweet, bitter and umami) [17] depend on GPCRs, as does chemosensation in nematodes. Vertebrate olfactory receptors generally conform to the one receptor type per Olfactory Sensory Neuron model [18], [19]. Mammalian sweet and umami taste receptors function as heterodimers [20], [21]. Nematode olfactory receptors pose an interesting dilemma because the genome of the free-living nematode, *C. elegans*, encodes >1300 GPCRs, many of which are believed to be chemoreceptors [22]. These receptors respond to many volatile odorants, but despite the large number of receptors, the nematode has only three pairs of olfactory neurons and only 32 pairs of chemosensory neurons in total [17], [23]. This inevitably requires that multiple types of chemoreceptor pairs are co-expressed in individual neurons [24], [25]. The question arises as to whether homo- or hetero-oligomerisation of nematode chemoreceptors can occur and if it does, whether it has any functional relevance in relation to chemoreceptor function, including ligand specificity.

So far, in *C. elegans*, only one GPCR, ODR-10, has been experimentally linked to olfaction. ODR-10 is a member of the *str* family of GPCRs, and is expressed predominantly in the AWA neuron pair [26], [27]. ODR-10 mediates chemotaxis towards diacetyl, a volatile ligand [27], [28]. We set out to investigate whether ODR-10 can form homo-oligomers or hetero-oligomers with other putative chemosensory GPCRs.

GPCR homo- and hetero-oligomers have previously been identified using various techniques such as bioluminescence resonance energy transfer (BRET), fluorescence resonance energy transfer (FRET), cross linking-studies and co-immunoprecipitation [29]–[32]. In this study we use the split-ubiquitin yeast two-hybrid system as well as BRET and co-immunoprecipitation to investigate potential homo- and hetero-oligomerisation of the *C. elegans* odorant receptor, ODR-10. We provide the first direct evidence that a nematode chemoreceptor (ODR-10) homo-oligomerises and can also form hetero-oligomers with STR-112, a nematode GPCR closely related by sequence, and the rat I7 odorant receptor, but not with the human somatostatin receptor 5 (SSTR5), a neuropeptide receptor. It is unknown whether such heteromerisation, in the distinctly unnatural environment of a yeast cell, is replicated or could have any functional significance *in vivo*.

## Materials and Methods

### Confocal microscopy

Yeast transformants expressing ODR-10-GFP^2^ were grown in selective dropout (SD) medium without histidine at 30°C overnight and the cells were inoculated into 10 ml of the same medium to give an initial absorbance at 600 nm (Abs_600_) = 0.025. The cells were again grown at 30°C overnight on a rotary shaker at 180 rpm. For microscopy, a 20 µL drop of yeast culture was placed on a clean glass slide and stained with 0.01% Evans Blue (Sigma) in culture medium, for 1min, covered with a cover slip and observed with a Leica SP2 confocal microscope (Germany) with 488 nm excitation, imaged at 510 nm for GFP^2^ emission and 660 nm for Evans Blue emission.

### Plasmid construction and transformation for immunoprecipitation and BRET

*Odr-10* was amplified by PCR from *C. elegans* cDNA prepared by standard techniques. Rat I7 receptor was amplified by PCR from the plasmid pCA4-I7-IRES-GFP, kindly provided by Prof S. Firestein [33]. SSTR5 receptor was amplified by PCR from the plasmid pGK-SSTR5-HA, kindly provided by Dr Jun Ishii [34]. The BRET tags Rluc and GFP^2^ were sourced from the plasmid pGFP^2^-MCS-Rluc(h) (Perkin Elmer) and introduced at the C-termini of *odr-10*, *I7* and *sstr5*, by PCR amplification as described previously [35]. All clones were verified by sequencing and cloned into pDONR201 (Invitrogen) using *att*B sites introduced by PCR. BRET-labelled odorant receptors were transferred into pYES-DEST52 (Invitrogen) or pDEST ESC-TRP by Gateway (Invitrogen) recombination for expression in *S. cerevisiae.* The vector pDEST ESC-TRP was constructed by introducing a 1.4 kb Gateway *att*R1-*ccd*B-*att*R2 cassette (Invitrogen) into the *Xho*I site of pESC-TRP (Stratagene), downstream of the *GAL1* promoter, using standard techniques. The plasmid pYES-DEST52 confers uracil selection in *S. cerevisiae* and pDEST ESC-TRP confers tryptophan selection. The two plasmids were transformed into *S. cerevisiae* strain InvSc1 (Invitrogen) either individually or in combination. Transformed yeast colonies were screened by PCR to confirm the presence of transgenes.

### Transformants culture and tagged-gene induction conditions

Yeast strains were transformed using the lithium acetate method [36]. Yeast was grown in a YPD medium [containing 1% yeast extract, 2% peptone and 2% glucose] or an SD medium [containing 0.67% yeast nitrogen base without amino acids and 2% glucose]. The SD medium was supplemented with appropriate amino acids depending on the selectable marker used. For solid media, 2% agar was added to the liquid media described above. In order to induce expression from the *GAL1* promoter, glucose was replaced with 1% raffinose and 2% galactose.

After overnight culture at 28°C, Abs_600nm_ was determined and cells were resuspended in induction medium to a final Abs_600nm_ of 0.4. This culture was incubated for varying times, with shaking at 15°C, to induce expression of receptor fusions. To test whether interactions between tagged receptors were specific or simply due to collisional interactions at high receptor density, Rluc and GFP^2^ labelled receptors were co-expressed in InvSc1 and induced for different times (0, 4, 8, 16, 24, 48 and 72 hrs) in order to generate total fluorescence levels up to 12-fold higher than untransformed cells. Total cell fluorescence was measured at different induction times in 100 µl (12.5 fold concentrate of cell sample with Abs_600nm_ = 0.4). Induction was stopped once the total fluorescence of the cells reached 1.2–2 fold higher than untransformed cells. Induced samples were pelleted and washed twice with cold phosphate-buffered saline (PBS). Induced cell suspensions were frozen as 12.5 fold concentrates in PBS, stored at −80°C and thawed in ice before assays.

### Immunoprecipitation assay

Cell lysates were prepared as described previously [35] using a French Press (∼18000 psi) in buffer B [75 mM tris-HCl, pH7.4, 12 mM MgCl_2_ and 2 mM EDTA, protease inhibitor cocktail *EASYpack* (Roche Applied Science)]. Cellular debris was removed by centrifugation at 15,000 rpm for 15 mins at 4°C and membranes were collected by ultracentrifugation of the supernatant at 40,000 rpm for 60 min at 4°C. Pellets containing membranes were re-suspended in ice-cold buffer B and left at 4°C overnight to resuspend completely. Membrane suspensions were solubilised using 1% digitonin in buffer B with a detergent/protein ratio of approximately 1∶1. The mixture was mixed gently for 3 hrs at 4°C and then centrifuged at 18,000 g for 30 mins at 4°C to remove insoluble material. The digitonin concentration was adjusted to 0.2%. An equivalent number of cells were processed for all immunoprecipitation reactions.

Immunoprecipitation procedures were modified from [37]. The luciferase activity of each sample was measured and reactions were standardised to equal luciferase activity. Polyclonal anti-green fluorescence protein antibody from rabbit (Sapphire Bioscience) was added to a final concentration of 0.001% (v/v) of serum. Immune complex formation was allowed to proceed overnight at 4°C with gentle agitation. Protein A-agarose (10% (w/v) Sigma) was added prior to a further 6 hr incubation. The agarose beads were sedimented by centrifugation at 5000 g for 3 mins at 4°C and were washed four times with ice-cold buffer B containing 0.2% digitonin. The luciferase activity of the beads was assessed using 5 µM coelenterazine H as substrate and an emission filter of 475±30 nm. Three independent yeast transformants were picked and each was processed and measured in triplicate. Data are presented as the mean percentage of maximal luciferase activity across all nine measurements.

### Quantification of yeast cells for BRET^2^ assay

All yeast cultures were adjusted to the same density (100 µl/well of Abs_600nm_ = 0.4, typically requiring 25 ml of culture resuspended in 2 ml of PBS buffer). All yeast strains expressing either receptor-GFP^2^, or receptor-Rluc and receptor-GFP^2^, fusion proteins were quantified by measuring the GFP^2^ fluorescence of 100 µl of Abs_600nm_ = 0.4. GFP^2^ fluorescence was measured in a SpectraMax M2 spectrofluorometer (Molecular Devices), with excitation centred at 420 nm and an emission filter of 510 nm, in a 96-well white Optiplate (Perkin Elmer). Fluorescence was expressed relative to the background determined in wells containing the same number of untransformed cells. Luminescence was measured by adding Coelenterazine H to a final concentration of 5 µM, using an emission filter of 475 nm in a lumino/fluorometer microplate reader (Polarstar Optima, BMGLabtech).

### Microplate BRET^2^ cell based assay

The assay, modified from Issad and Jockers [38], was conducted in duplicate in a 96-well white Optiplate in a total volume of 100 µl. Three independent transformants were processed and the data were pooled. Coelenterazine (DeepBlueC, Biosynth AG) was added to a final concentration of 10 µM, and readings were performed using a Polarstar Optima (BMGLabtech). Negative controls were: the host strain expressing ODR-10-Rluc/SSTR5-GFP^2^; the host strain expressing Rluc/GFP^2^; a mixture of the strain expressing ODR-10-Rluc only and the strain expressing ODR-10-GFP^2^ only. Rluc light emission was measured at 410±80 nm and GFP^2^ light emission at 515±30 nm. The BRET^2^ ratio is defined as the ratio of the emission intensities at 515 nm and 410 nm. Subtraction of the auto-fluorescence from all tested samples was undertaken before BRET ratio values were calculated.

### Protein-protein interaction assays using split-ubiquitin membrane yeast two-hybrid system

The split-ubiquitin system [39] was used to investigate the interactions among ODR-10, I7, SSTR5 and STR-112 GPCRs. Vectors and yeast strain were as supplied in the DUALmembrane pairwise interaction kit (Dualsystems Biotech, Zürich, Switzerland). Full-length cDNAs of ODR-10, I7 and STR-112 were cloned into the pBT3-STE plasmid encoding the C-terminal half of ubiquitin (Cub) such that it was fused to the C-terminus of the GPCR and pPR3-STE encoding the mutated N-terminal half of ubiquitin (NubG) such that it was fused to the C-terminus of the GPCR. All cDNAs were cloned into *Sfi* I restriction sites. cDNA sequences were confirmed by DNA sequencing. Cub and NubG fusion constructs were co-transformed into the yeast strain NMY51. Interaction was determined by assessing the growth of yeast transformants on medium lacking histidine and confirmed by β-galactosidase assay (HTX Kit: Dualsystems Biotech, Zürich, Switzerland).

## Results and Discussion

### Localisation of nematode GFP^2^ fluorescent-tagged ODR-10 in yeast

We chose to express nematode receptor constructs in *Saccharomyces cerevisiae* because there is extensive literature showing that yeast can be used to functionally express GPCRs [40]–[42], including the nematode chemoreceptor ODR-10 [35].

**Figure 1:**
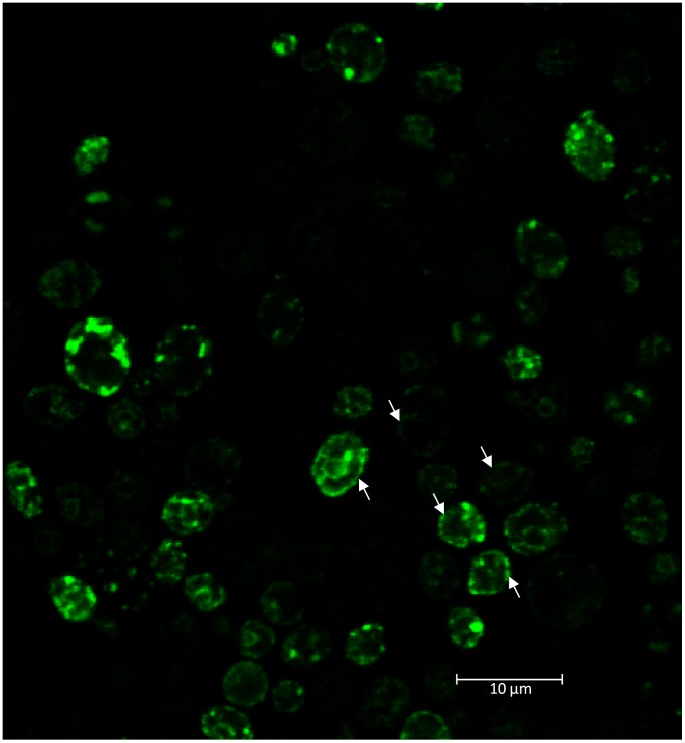
Yeast cells expressing ODR-10-GFP^2^. Arrows indicate the plasma membrane and cytoplasmic localisation. Bar: 11.9 µm. Image was obtained with a Leica SP2 confocal laser scanning microscope using excitation at 488 nm.

To observe the subcellular localisation of the ODR-10 receptor in yeast, a GFP^2^ fluorescent tag was introduced at its C-terminus. Yeast cells expressing ODR-10-GFP^2^ were examined by confocal microscopy. In yeast cells co-expressing ODR-10-GFP^2^ and ODR-10-Rluc, ODR-10-GFP^2^ was localised to the plasma membrane in ≈32% of cells after a 4 hour induction (Figure 1). Although some of ODR-10 was localised to the yeast plasma membrane, much of it was localised intracellularly (Figure 1), which has also previously been observed for mammalian chemoreceptors [34], [43], [44]. However, it has also been shown that mammalian olfactory receptors are functional whether they are localised to yeast ER, Golgi or plasma membrane when tested *in vitro* [34], [43], [44]. Based on these previous reports, we believe it is reasonable to infer that ODR-10 is correctly folded whether it is located in the plasma or intracellular membranes [35].

### ODR-10 forms homo-oligomers

**Figure 2:**
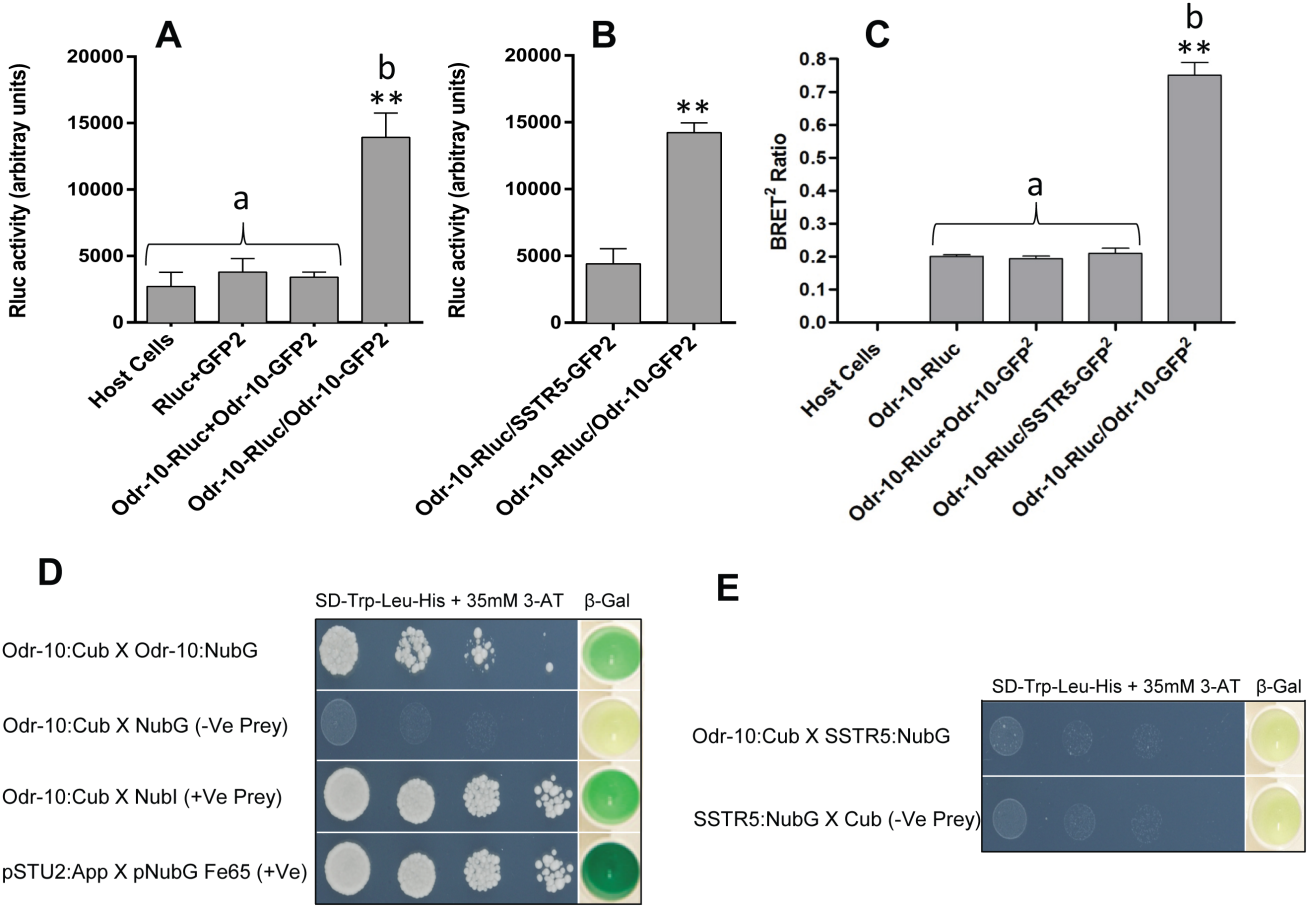
Three lines of evidence for formation of ODR-10 homo-oligomers. **A.** Co-immunoprecipitation of a pair of tagged chemoreceptor subunits. **B.** ODR-10-Rluc co-expressed with SSTR5-GFP^2^ as a negative control. Following membrane extraction with digitonin and immunoprecipitation with an anti-GFP^2^ antibody, as described in Materials and Methods, luciferase activity was measured using 5 µM Coelenterazine H with an emission filter of 475±30 nm. Values represent means ± SD of experiments performed on three independent transformants (**denotes significance at P≤0.001), “b” is significantly different from “a”. **C.** BRET^2^ ratios measured to test for homo-dimerisation of ODR-10 in intact yeast cells. Values represent means ± SD (n = 3) (**denotes significance at P≤0.001), “b” is significantly different from “a”. Indicated constructs in both **A** and **C** were expressed separately, or mixed (+), or co-expressed (/). **D.** ODR-10 homo-oligomerises in the split-ubiquitin yeast two-hybrid system. The C-terminal half of ubiquitin (Cub) was fused to the C-terminus of the full-length cDNA of *odr-10* **(ODR-10::Cub)**. The N-terminal half of ubiquitin (NubG) was fused to the C- termini of full-length cDNA of *odr-10* **(ODR-10::NubG)**. The interaction of ODR-10**::**Cub with pOST1:NubI served as a positive control to ensure the correct topology of the fusion protein. The interaction of ODR-10::Cub with the empty vector (pPR3-STE) served as a negative control. Yeast transformants containing both a Cub fusion and a NubG fusion construct were grown on drop out media (SD -Leu and -Trp) (Figure S2A) and selective medium lacking histidine (SD -Leu, -Trp and -His) containing 35 mM 3-Amino-1, 2, 4-triazole (3-AT). β-galactosidase assays were performed to verify interactions. Cells were spotted as one-tenth dilutions starting at Abs_600nm_ = 1. **E.** ODR-10 does not hetero-oligomerise with SSTR5 in the split-ubiquitin yeast two-hybrid system. Growth and β-galactosidase activity of yeast cells expressing ODR-10:Cub and SSTR5:NubG fusions. The interaction of SSTR5::NubG with the empty vector (pBT3-STE) served as a negative control. Yeast transformants containing both a Cub fusion and a NubG fusion constructs were grown on drop out media (SD -Leu and -Trp) (Figure S2E) and selective medium lacking histidine (SD -Leu, -Trp and -His) containing 35 mM 3-Amino-1, 2, 4-triazole (3-AT). β-Galactosidase assays were performed to verify interactions. Cells were spotted as one-tenth dilutions starting at Abs_600nm_ = 1.

We tested for homo-oligomerisation of ODR-10 using three different methods; co-immunoprecipitation, bioluminescence resonance energy transfer (BRET) and the split-ubiquitin yeast two-hybrid system. Co-immunoprecipitation has been used extensively as a biochemical test for GPCR oligomerisation [45]–[50]. We co-expressed an Rluc-tagged receptor (ODR-10-Rluc) and a GFP^2^-tagged receptor (ODR-10-GFP^2^). Rluc-labelled ODR-10, co-expressed with GFP^2^-labelled ODR-10 was pulled down by anti-GFP antibody, as evidenced by a high level of co-immunoprecipitated luciferase activity (Figure 2A). Control immunoprecipitates containing untransformed yeast cells, Rluc co-expressed with GFP^2^, or a mixture of separately expressed ODR-10-GFP^2^ and ODR-10-Rluc, all showed approximately 20% of the total luminescence of the co-expressed receptor pairs (Figure 2A). We also co-expressed ODR-10-Rluc with SSTR5-GFP^2^, as a negative control, and this also showed approximately 20% of the total luminescence of the co-expressed receptor pairs (Figure 2B). This is the first evidence that ODR-10 or any nematode GPCR can form homo-oligomers.

To determine whether receptor oligomerisation also occurs in intact yeast cells, we used bioluminescence resonance energy transfer (BRET). BRET has previously been used to investigate GPCR oligomerisation in living cells. BRET^2^ gives a clearer separation than standard BRET between the emission spectra of Renilla luciferase (Rluc) and the green fluorescent protein (GFP^2^), resulting in much improved signal to noise ratio at the expense of a lower quantum efficiency [35], [51], [52]. To compensate for the lower luminescence, we used high-copy number plasmids to express tagged ODR-10 variants under the *GAL1* inducible promoter. By monitoring GFP intensity of the transformed cells, we generated a series of samples in which GFP expression levels sequentially increased over background with increasing induction times. BRET^2^ experiments were conducted at GFP^2^ levels no more than twofold greater than background, in order to minimise BRET^2^ due to possible non-specific interactions between ODR-10-Rluc and ODR-10-GFP^2^, so called “bystander BRET”, due to overexpression (Figure S1).

BRET^2^ results supported our co-immunoprecipitation conclusions and show that ODR-10 fusion proteins can form homo-oligomers in living yeast cells (Figure 2C). The BRET^2^ ratio was 0.750±0.038 in yeast cells co-expressing ODR-10-Rluc and ODR-10-GFP^2^, which was statistically significantly different (P<0.001) from samples, co-expressing ODR-10-Rluc/SSTR5-GFP^2^ (BRET^2^ signal, 0.209±0.012) and the mixture of cells separately expressing ODR-10-Rluc and ODR-10-GFP^2^ (BRET^2^ ratio 0.193±0.007). Cells expressing neither GFP^2^ nor Rluc had no measurable BRET^2^ ratio. The Forster distance of the BRET^2^ system is approximately 7.5 nm [53], which means that interacting proteins have to bring the RLuc and GFP^2^ tags within about 10 nm of each other and be suitably aligned to see an appreciable change in the signal.

**Figure 3:**
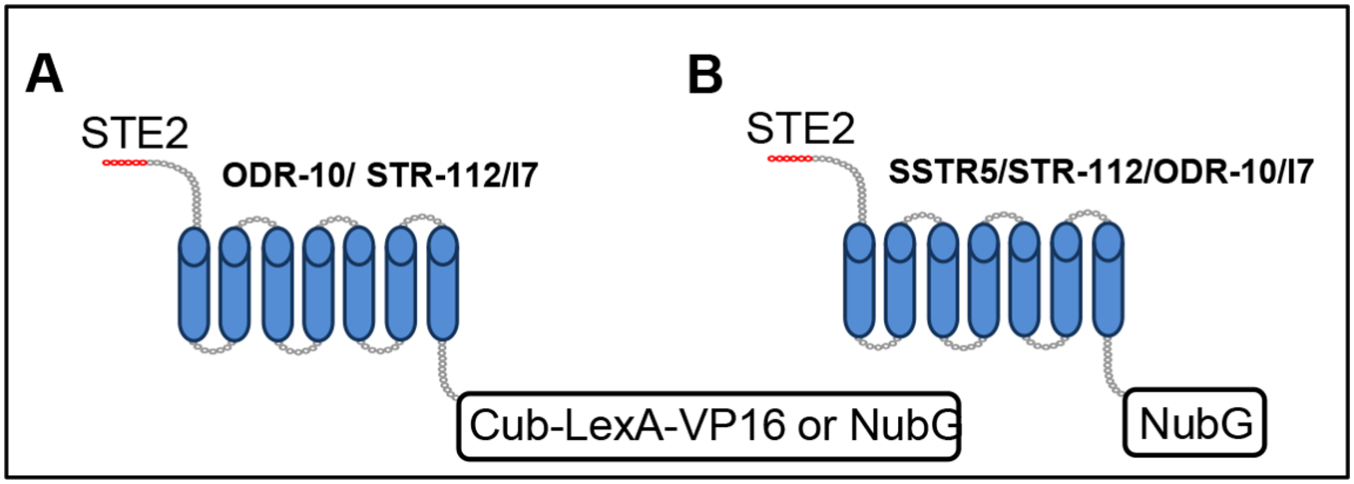
Constructs used for split-ubiquitin assays. **A.** Bait fusion proteins were made with the C-terminus of ubiquitin (Cub) linked to the synthetic transcription factor LexA-VP16 at the C-termini of ODR-10, STR-112 and I7 in the pBT3-STE plasmid. The STE2 sequence improves translation of protein constructs. **B.** Prey protein fusions were constructed with the mutant N-terminal part of ubiquitin (NubG) at the C termini of the SSTR5, STR-112, ODR-10 and I7. If bait and prey interact, NubG and Cub are brought into close proximity, resulting in the reconstitution of functional ubiquitin and release of the LexA-VP16 transcriptional factor, which leads to transcriptional readout, resulting in growth of host yeast cells on the selective medium (SD-L-T-H) or color development in a β-galactosidase assay.

As a third, independent, test for the homo-oligomerisation of ODR-10, we used a split-ubiquitin yeast two-hybrid system, which has been developed to assess interactions between membrane proteins [39], [54]–[58]. In this system, proteins of interest are fused to either the N- or C-terminal moiety of a mutated ubiquitin (Figure 3). The N-terminus of split-ubiquitin includes an Ile to Gly mutation (NubG), which abolishes NubG’s affinity for Cub when expressed in the same cells. The C-terminal moiety of the split-ubiquitin includes an artificial transcription factor domain (Cub-LexA-VP16). Upon *in vivo* interaction of their respective protein fusion partners, NubG and Cub are forced into close proximity and the interaction can be detected by the release of LexA-VP16 transcription factor, inducing transcriptional activation of growth and colorimetric reporter genes (*HIS3*, *ADE2*, and *lacZ*). We used this system to test for oligomerisation of nematode ORs.

The SignalP 3.0 algorithm predicts that *odr-10* does not contain a cleavable N-terminal sequence [59]. Therefore, we fused full-length *odr-10* to the C-terminus of ubiquitin in pBT3-STE (ODR-10:Cub) and also to the N-terminus of ubiquitin in pPR3-STE (ODR-10:NubG). The STE2 sequence improves translation of bait and prey sequences. In order to test the homo-oligomerisation of ODR-10, both constructs were co-transformed into the reporter yeast strain (NMY51). In the presence of 35 mM 3-Amino-1, 2, 4-triazole (3-AT), which is required to establish an appropriate level of stringency, ODR-10-Cub interacted with ODR-10-NubG, shown by the growth of this pair on drop out media lacking His and confirmed by β-galactosidase assay (Figure 2D). A negative control expressing ODR-10-Cub with NubG alone (pPR3-STE) showed no growth (Figure 2D). Co-expression of ODR-10-Cub with wild-type Nub I (Ost1-NubI) served as a positive control, which resulted in growth on His-deficient medium and expression of the *lacZ* reporter as shown in Figure 2D. Growth of yeast expressing ODR-10-Cub and the Ost1-NubI control also confirms that ODR-10 is functional in this yeast system. The bait pTSU2-APP, expressing the type I integral membrane protein amyloid A4 precursor protein (APP) and the prey pNubG-Fe65 expressing the cytosolic protein amyloid beta A4 precursor protein-binding family B member 1 (Fe65) were used as positive controls (Figure 2D). These results, obtained using three independent approaches, provide the first evidence that a nematode chemoreceptor can form homo-oligomers.

### ODR-10 can form hetero-oligomers with other olfactory receptors but not with SSTR5

**Figure 4:**
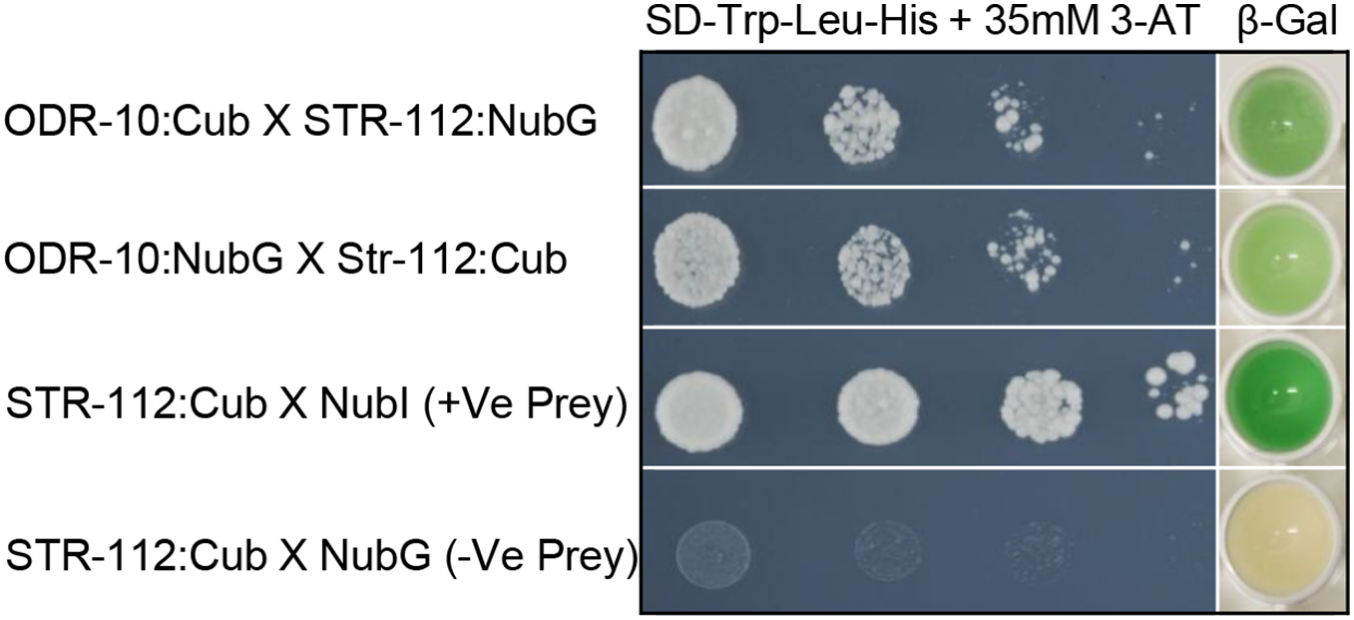
ODR-10 hetero-oligomerises with STR-112 in the split-ubiquitin yeast two-hybrid system. Growth and β-Galactosidase activity of yeast cells expressing various combinations of Cub and NubG fusions. The control plasmids were pPR3-STE and pBT3-STE. Yeast transformants containing both Cub fusion and NubG fusion constructs were grown on drop out media (SD -Leu and -Trp) (Figure S2B) and selective medium lacking histidine (SD -Leu, -Trp and -His) containing 35 mM 3-Amino-1, 2, 4-triazole (3-AT). β-galactosidase assays were performed to verify interactions. Cells were spotted as one-tenth dilutions starting at Abs_600nm_ = 1.

To investigate whether ODR-10 can form hetero-oligomers with other GPCRs, we probed the interaction of ODR-10 with a second *C. elegans* chemoreceptor, STR-112 and two mammalian receptors, the rat I7 chemoreceptor [33], [60] and the human somatostatin receptor subtype 5 (SSTR5) [34], [61]. STR-112 is the closest homologue of ODR-10, having 79.6% amino acid identity with it. We used the split-ubiquitin yeast two-hybrid system to test for hetero-oligomerisation of ODR-10 with STR-112. Thus, full-length *str-112* was fused to the C-terminus of ubiquitin in pBT3-STE (STR-112:Cub) and also to the N-terminus of ubiquitin in pPR3-STE (STR-112:NubG) as shown in Figure 3. Pairwise interaction was tested, in both ODR-10-Cub/STR-112-NubG and STR-112-Cub/ODR-10-NubG combinations. As shown in Figure 4, ODR-10 interacted with STR-112, demonstrated by growth on drop out medium lacking His as well as by a β-galactosidase induction assay. No growth was observed in negative controls, under the same conditions (Figure 4).

**Figure 5:**
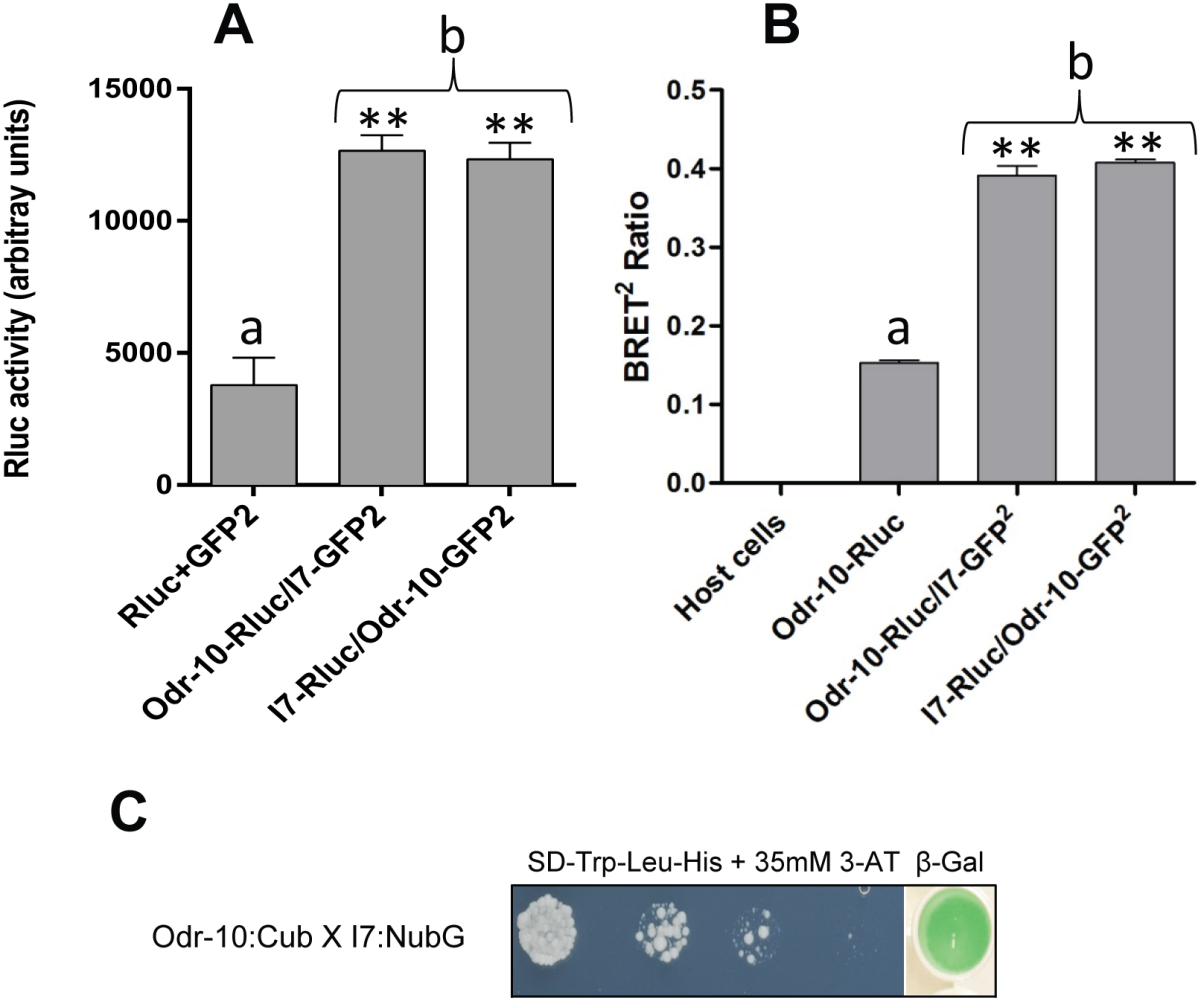
ODR-10 hetero-oligomerises with I7. **A.** Co-immunoprecipitation of pairs of tagged chemoreceptor subunits. Values represent means ± SD of experiments performed in triplicate with three independent transformants for each condition (**denotes significance at P≤0.001), “b” is significantly different from “a”. **B.** BRET^2^ ratios measured to test for hetero-dimerisation of ODR-10 and I7 in intact yeast cells. Values represent means ± SD (n = 3) (**denotes significance at P≤0.002), “b” is significantly different from “a”. Indicated constructs in both **A** and **B** were expressed separately, or mixed (+), or co-expressed (/). **C.** ODR-10 hetero-oligomerises with I7 in the split-ubiquitin yeast two-hybrid system. Growth and β-galactosidase activity of yeast cells expressing ODR-10:Cub and I7:NubG fusions. Yeast transformants containing both Cub fusion and NubG fusion constructs were grown on drop out media (SD -Leu and -Trp) (Figure S2C) and selective medium lacking histidine (SD -Leu, -Trp and -His) containing 35 mM 3-Amino-1, 2, 4-triazole (3-AT). β-galactosidase assays were performed to verify interactions. Cells were spotted as one-tenth dilutions starting at Abs_600nm_ = 1.

**Figure 6:**
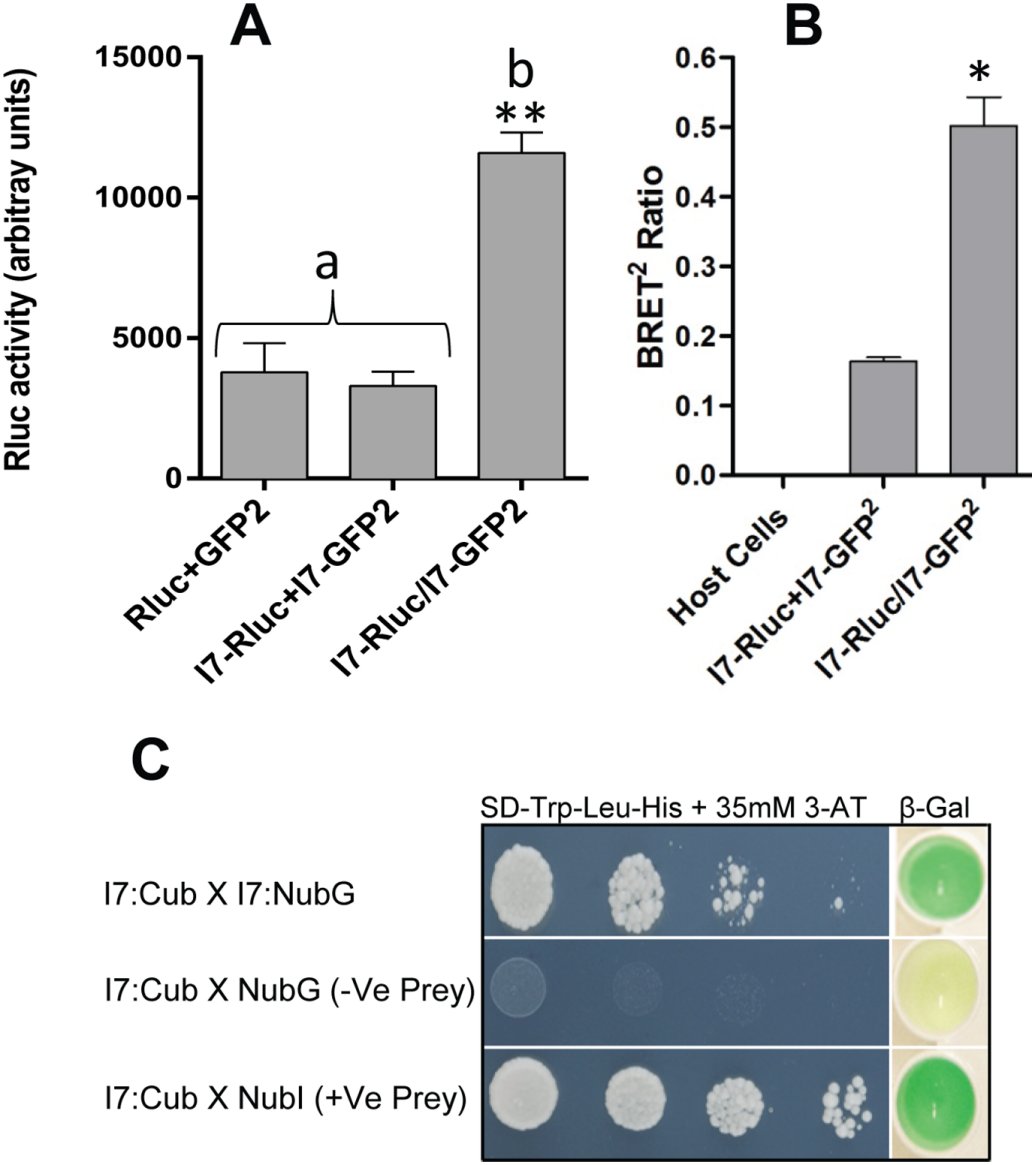
I7 forms homo-oligomers. **A.** Co-immunoprecipitation of a pair of tagged chemoreceptor subunits. Values represent means ± SD of experiments performed in triplicate with three independent transformants for each condition (**denotes significance at P≤0.001), “b” is significantly different from “a”. **B.** BRET^2^ ratios measured to test for homo-oligomerisation of I7 in intact yeast cells. Values represent means ± SD of experiments performed in triplicate with three independent transformants for each condition. (*denotes significance at P≤0.01). Indicated constructs in both **A** and **B** were expressed separately, or mixed (+), or co-expressed (/). **C.** I7 homo-oligomerises in the split-ubiquitin yeast two-hybrid system. Growth and β-galactosidase activity of yeast cells expressing I7:Cub and I7:NubG fusions. The interaction of I7::Cub with the empty vector (pPR3-STE) served as a negative control. Yeast transformants containing both a Cub fusion and a NubG fusion constructs were grown on drop out media (SD -Leu and -Trp) (Figure S2D) and selective medium lacking histidine (SD -Leu, -Trp and -His) containing 35 mM 3-Amino-1,2,4-triazole (3-AT). β-galactosidase assays were performed to verify interactions. Cells were spotted as one-tenth dilutions starting at Abs_600nm_ = 1.

Immunoprecipitation experiments also showed that rat I7 can form heterodimers with ODR-10 (Figure 5A). We confirmed hetero-oligomerisation between ODR-10 and I7 using two other, independent, techniques. Co-expression of ODR-10-Rluc with I7-GFP^2^ or ODR-10- GFP^2^ with I7-Rluc gave BRET^2^ ratios of 0.39±0.12 and 0.40±0.004 respectively, compared with a BRET^2^ ratio of 0.15±0.004 for ODR-10-Rluc alone, indicating that ODR-10 heterodimerises with I7 (Figure 5B). Furthermore, split-ubiquitin yeast two-hybrid experiments confirmed hetero-oligomerisation of ODR-10 and I7. Pairwise interaction was performed using ODR-10-Cub and I7-NubG combination. As shown in Figure 5C, ODR-10 interacted with I7, observed by the growth on drop out medium lacking His as well as β-galactosidase assay. Immunoprecipitation experiments showed that I7 itself can form homo-oligomers (Figure 6A). Co-expression of I7-Rluc with I7-GFP^2^ gave a BRET^2^ ratio of 0.50±0.04 compared with 0.016±0.006 for a post-expression mixture (Figure 6B), which is statistically significant (P≤0.01). Furthermore, rat I7 scored positive for homo-oligomerisation in the split-ubiquitin yeast two-hybrid assay (Figure 6C) both by growth on drop out media lacking His, and by β-galactosidase assay. This is consistent with the finding of Wade et al [62], that hOR1740, a human helional receptor, can form functional homodimers.

Given the evolutionary distance separating nematodes and mammals and their odorant receptors [63], we were somewhat surprised that ODR-10 and I7 interacted It seems plausible, although we have not shown it, that ODR-10 oligomers are functional. It is also conceivable that ODR-10 may form functional oligomers with other nematode ORs, such as STR-112. However, ODR-10 and I7 are never co-expressed in nature and their oligomerisation could only be meaningful in a structural, rather than a functional sense.

We therefore also used the yeast two-hybrid system to test whether ODR-10 can interact with a more distantly related, non-olfactory, mammalian GPCR. For this purpose we chose the human somatostatin receptor, SSTR5, a class A GPCR. We tested pairwise interaction between ODR-10-Cub and SSTR5-NubG using the split-ubiquitin yeast two-hybrid system. As shown in Figure 2E, ODR-10 did not interact with SSTR5, demonstrated by lack of growth on drop out media lacking His, and lack of β-galactosidase expression. It is possible that the enhanced BRET signals observed, whilst not being completely non-specific, only occur at the receptor concentrations achieved in the heterologous yeast system. It remains to be seen whether such interactions can be observed in the natural environment of a nematode OSN.

## Conclusion

Three independent lines of evidence show that, in the heterologous yeast system, the *C. elegans* olfactory receptor ODR-10 forms homo-oligomers. Homo-oligomerisation has been previously described for mammalian olfactory receptors [62]. ODR-10 can also oligomerise with a closely related *C. elegans* chemosensory receptor, and even with a mammalian olfactory receptor. In contrast, we saw no evidence for hetero-oligomerisation between ODR-10 and the human peptide receptor SSTR5. However, we cannot rule out the possibility that ODR-10 might interact with some non-chemosensory GPCRs. One previous study found that a single mammalian olfactory receptor, screened indirectly for interactions with 42 distinct GPCR subtypes, interacted with only three purinergic receptors [64]. These interactions appeared to be receptor specific, as other olfactory receptors tested did not interact with the purinergic receptors. Vertebrate olfactory neurons typically express only one or two receptors per neuron [18], [19]. In these systems, perception of odours is processed in higher order brain centres by simultaneously analysing the inputs of thousands of neurons [65]. *C. elegans* has hundreds of chemoreceptor genes, but only 32 chemosensory neurons, implying that a single neuron expresses many different chemoreceptor genes [24], [66]. Our observations raise the possibility that *C. elegans* chemosensory receptors expressed in the same sensory neuron *in vivo* could form heterodimers, which would have the potential to increase the functional repertoire of individual OSNs. However, we emphasise that we have no evidence that such interactions occur *in vivo*, nor that they are functional even in the yeast system used here. It would be interesting to search for hetero-oligomerisation *in vivo*, by transforming *C. elegans* with neuronally-targetted pairs of BRET-tagged odorant receptors.

## Supporting Information

Figure S1 BRET^2^ results showed that specific signals obtained from a series of low level expression of tagged ODR-10 samples showed that interactions between ODR-10 were specific and not collisional interactions due to over-expression. Tested yeast cells were induced for expressing tagged ODR-10 proteins for different times (between 0 to 72 hours) at 15°C in order to achieve different levels of ODR-10 expression as indicated by tagged GFP levels from 1 to 12-fold over the untransformed cells. Only samples with GFP^2^ levels≤2 fold greater than controlled cells (to the left of the vertical line) were used for oligomerisation studies. Energy transfer measurements were performed in living cells by adding 5 µM DeepBlueC and measured light emissions in a dual wavelength microplate reader with Rluc and GFP filter settings as described in the Methods. Values represent means ± SD of two independent experiments.
(TIF)

Figure S2 Positive controls for Figures 2, 4, 5 and 6. Yeast transformants containing both a Cub fusion and a NubG fusion construct were grown on drop out media (SD -Leu and -Trp) to test the presence of both constructs in yeast cells. Cells were spotted as one-tenth dilutions starting at Abs_600nm_ = 1.
(TIF)
