# Supplementary figures and images for: Oligomerisation of C. elegans Olfactory Receptors, ODR-10 and STR-112, in Yeast
Source: PLoS One. 2014 Sep 25;9(9):e108680. doi: 10.1371/journal.pone.0108680 (PMC4177895; doi:10.1371/journal.pone.0108680)

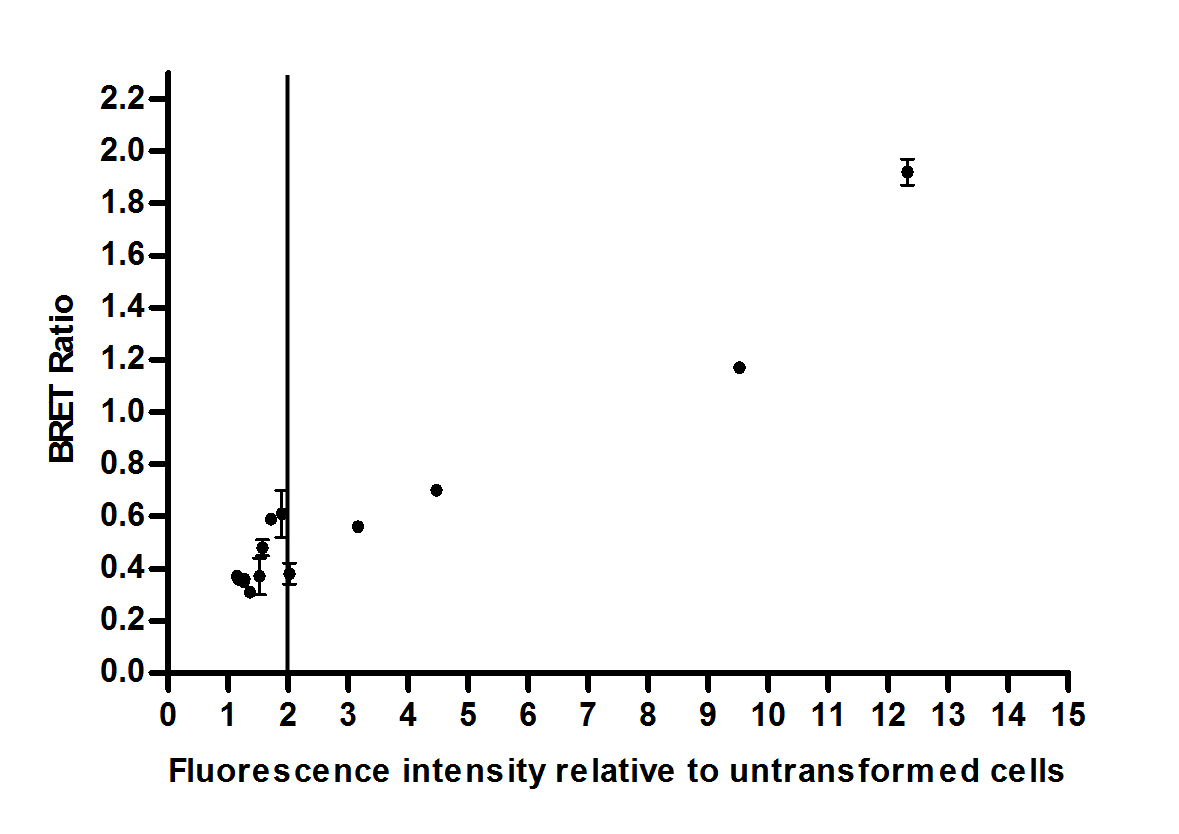

Supplement: Figure S1 — BRET2 results showed that specific signals obtained from a series of low level expression of tagged ODR-10 samples showed that interactions between ODR-10 were specific and not collisional interactions due to over-expression. Tested yeast cells were induced for expressing tagged ODR-10 proteins for different times (between 0 to 72 hours) at 15°C in order to achieve different levels of ODR-10 expression as indicated by tagged GFP levels from 1 to 12-fold over the untransformed cells. Only samples with GFP2 levels≤2 fold greater than controlled cells (to the left of the vertical line) were used for oligomerisation studies. Energy transfer measurements were performed in living cells by adding 5 µM DeepBlueC and measured light emissions in a dual wavelength microplate reader with Rluc and GFP filter settings as described in the Methods. Values represent means ± SD of two independent experiments. (TIF) [file pone.0108680.s001.tif]

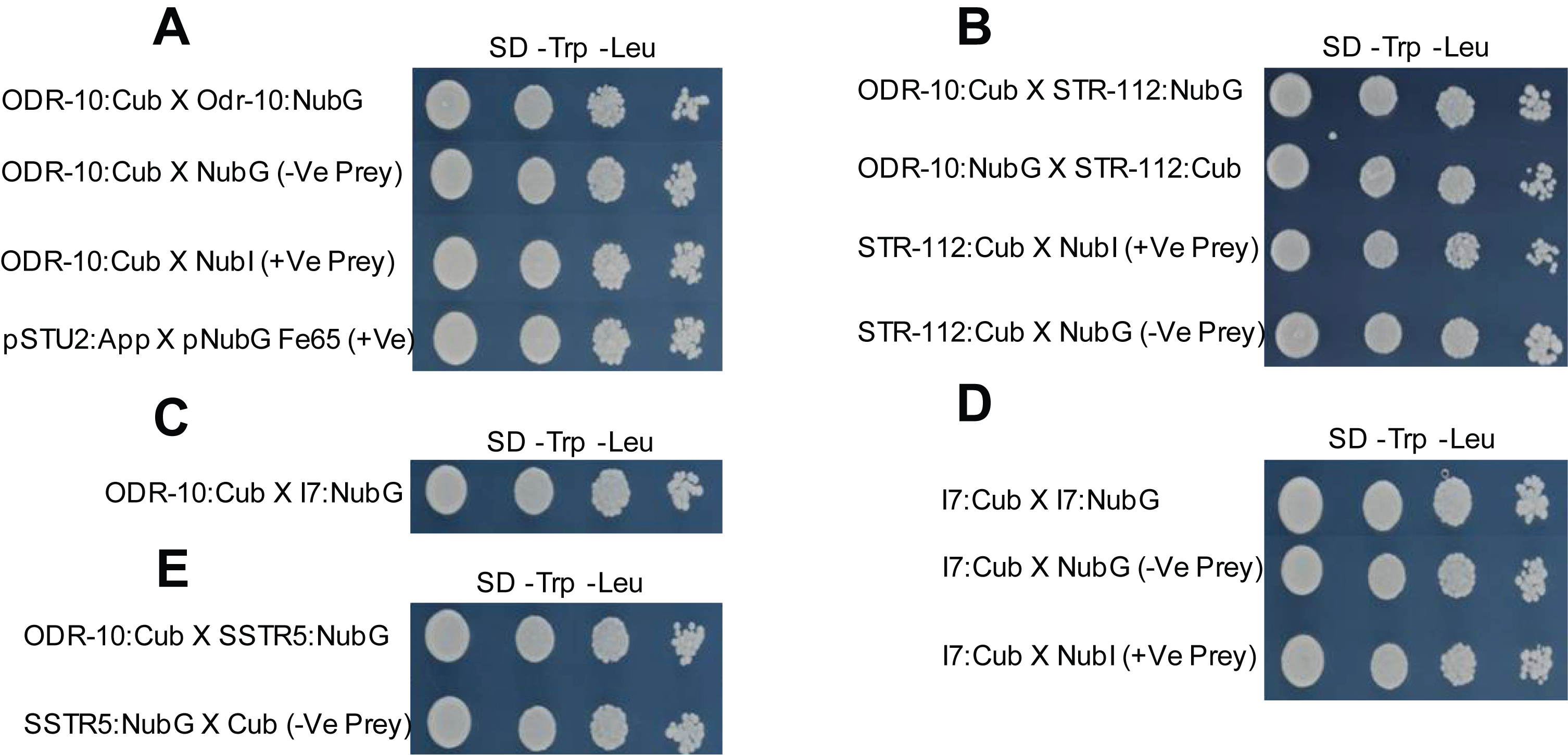

Supplement: Figure S2 — Positive controls for Figures 2, 4, 5 and 6. Yeast transformants containing both a Cub fusion and a NubG fusion construct were grown on drop out media (SD -Leu and -Trp) to test the presence of both constructs in yeast cells. Cells were spotted as one-tenth dilutions starting at Abs600nm = 1. (TIF) [file pone.0108680.s002.tif]
